# Fabrication of a Molybdenum Dioxide/Multi-Walled Carbon Nanotubes Nanocomposite as an Anodic Modification Material for High-Performance Microbial Fuel Cells

**DOI:** 10.3390/molecules29112541

**Published:** 2024-05-28

**Authors:** Jianchun Ma, Lifang Wang, Yezhen Zhang, Jianfeng Jia

**Affiliations:** 1 Department of Chemical and Material Engineering, Lyuliang University, Lishi 033001, China; lllswlf@163.com; 2 Institute of New Carbon-Based Materials and Zero-Carbon and Negative-Carbon Technology, Lyuliang University, Lishi 033001, China; 3 College of Chemistry and Pharmacy Engineering, Nanyang Normal University, Nanyang 473061, China; zhangyezhenfang@sina.com; 4 Key Laboratory of Magnetic Molecules and Magnetic Information Materials of Ministry of Education, School of Chemistry and Materials Science, Shanxi Normal University, Taiyuan 030031, China

**Keywords:** molybdenum dioxide nanoparticle–decorated multi-walled carbon nanotubes nanocomposites, microbial fuel cells, anodic modification materials, power density

## Abstract

A nanocomposite of multi-walled carbon nanotubes (MWCNTs) decorated with molybdenum dioxide (MoO_2_) nanoparticles is fabricated through the reduction of phosphomolybdic acid hydrate on functionalized MWCNTs in a hydrogen–argon (10%) atmosphere in a tube furnace. The MoO_2_/MWCNTs composite is proposed as an anodic modification material for microbial fuel cells (MFCs). MWCNTs have outstanding physical and chemical peculiarities, with functionalized MWCNTs having substantially large electroactive areas. In addition, combined with the exceptional properties of MoO_2_ nanoparticles, the synergistic advantages of functionalized MWCNTs and MoO_2_ nanoparticles give a MoO_2_/MWCNTs anode a large electroactive area, excellent electronic conductivity, enhanced extracellular electron transfer capacity, and improved nutrient transfer capability. Finally, the power harvesting of an MFC with the MoO_2_/MWCNTs anode is improved, with the MFC showing long-term repeatability of voltage and current density outputs. This exploratory research advances the fundamental application of anodic modification to MFCs, simultaneously providing valuable guidance for the use of carbon-based transition metal oxide nanomaterials in high-performance MFCs.

## 1. Introduction

The ever-increasing global energy consumption requirements, involving the excessive depletion of unsustainable fossil fuel and consequent severe environmental pollution problems, are intensively driving the development of green, renewable carbon-neutral energy technologies [1,2,3,4,5,6]. Microbial fuel cells (MFCs), utilizing effectual electroactive bacteria as anodic electricigens, can directly transform the chemical energy of inexhaustible organic matter or biomass into available clean electrical energy [7,8,9,10,11,12]. MFCs can effectively remove environmental waste, such as wastewater, while producing bioelectricity [13]. This feature is considerably attractive to scientific researchers in the MFC field worldwide.

Despite the excellent bioelectricity generation and breakthrough of MFCs in the past few decades [14,15,16,17], their low power harvesting and high manufacturing cost inputs immensely restrict their practical use [18,19,20,21,22]. Many factors can influence the power generation performance of MFCs, such as cell configurations, anodic or cathodic modification materials, proton exchange membranes, anolytes, and electron media [4,23,24,25,26]. Anodic modification plays an essential role in MFC performance, and diversifying anodic modification materials through exploration is a valuable research direction in the MFC field.

Carbon nanotubes (CNTs), which are one-dimensional carbon nanomaterials, have excellent physical and chemical peculiarities due to their special structural characteristics. CNTs have been utilized as anodic modification materials of MFCs due to their excellent electronic conductivity, mechanical properties, and biocompatibility [27]. Additionally, numerous CNTs can efficaciously constitute three-dimensional network structures; such structures can form large holes and are conducive to the attachment and habitation of active microorganisms. Sun et al. [28] fabricated three-dimensional CNT networks on an MFC anode through layer-by-layer self-assembly. The modified anode with CNTs had a large specific surface area. The interface electron transfer impedance of MFCs with this anode reduced from 1163 to 258 Ω, and the power density increased by 20% compared to the bare carbon paper anode. Peng et al. [29] modified a glassy carbon electrode using CNT materials and discovered that CNTs can accelerate the extracellular electron transfer (EET) between the active microorganism *Shewanella oneidensis* and an anode. The current density output of the MFC was 9.70 ± 0.40 μA cm^−2^, which was 82-fold higher than that of an MFC with an unmodified anode. Liang et al. [30] directly mixed CNTs and the bacterium *Geobacter sulfurreducens* in a composite biofilm architecture and applied it as an MFC anode. The experimental results exhibit markedly reduced startup time and anode resistance of the MFC. The voltage output and electricity production performance of the MFC were also heightened. Moreover, the maximum power density of the MFC based on the CNT hydrogel biological anode was 65% higher than that of the control group [31]. However, for unfunctionalized CNTs with exiguous surface defects, some functional groups in the end caps of nanotubes, such as hydroxyls, cannot be completely exposed; these reduce the dispersibility of CNTs. As anodic modification materials, unfunctionalized CNTs have small active areas and are not conducive to the heavy exposure of catalytic sites [32]. CNT surface functionalization is usually performed using a strong oxidizer or acid corrosion, and various functional groups can be introduced into purified CNT surfaces. Through synergistic interaction with intercalation of concentrated sulfuric acid and oxidation of concentrated nitric acid, mixed-acid treatment can purify CNTs by removing their impurities; it can also connect large numbers of hydroxyl, carboxyl, and other oxygen-containing groups on CNT surfaces. This can enhance CNT dispersion and the binding of CNTs with other substances, enhancing the efficiency of decorating CNTs with metal nanoparticles or oxide particles [33].

Molybdenum minerals are abundant in nature and cost-effective. In recent years, transition metal compounds containing molybdenum have also garnered considerable attention. Molybdenum carbide (Mo_2_C), a transition metal carbide with a high melting point and hardness, has outstanding thermal stability, corrosion resistance, electronic structure, and catalytic activity, similar to precious metals [34,35,36]. Mo_2_C has been utilized as an anodic modification material for MFCs. Zou et al. [2] used Mo_2_C nanoparticles with small grain sizes and good crystallinity to modify porous graphene nanocomposites via electrostatic assembly combined with high-temperature carburization, greatly enhancing the adhesion of active bacteria and the allegro formation of stable biofilms. These active bacteria secreted adequate electrochemical biomolecules, such as flavin, around the anode, thus increasing the EET rate from the bacterial cells to the anode. Finally, the MFC proposed by Zou et al. achieved a satisfactory power density of 1697 mW m^−2^, which was twice and 13 times those of MFCs with graphene and bare carbon cloth (CC) anodes, respectively. MoO_2_ is also a transition metal oxide with a high melting point. MoO_2_ has superior conductivity, chemical stability, and charge transmission properties; thus, it has potential applications in catalysts, chemical sensors, supercapacitors, lithium-ion batteries, electrochromic displays, and field-emission materials [37]. The valence band of MoO_2_ has many high-density free electrons, which can effectively accelerate the catalytic activity of Mo and improve the catalytic performance of MoO_2_; therefore, MoO_2_ has been diffusely applied in the field of catalysis. Because of its outstanding conductivity and charge carrier transfer efficiency and the tunnel-like voids in its crystal structure, MoO_2_ is beneficial for embedding and extricating charged particles at high speeds; hence, MoO_2_ is a candidate material in the field of supercapacitors [38]. MoO_2_ has been utilized as an anodic modification material for MFCs. Zeng et al. [39] synthesized polydopamine-modified Mo_2_C/MoO_2_ nanoparticles through thermal reduction incorporating in situ polydopamine modification and recommended them for MFC anodic modification. The maximum power density of their MFC was 1.64 ± 0.09 W m^−2^. Li et al. [40] prepared Co-modified MoO_2_ nanoparticles dispersed on nitrogen-doped carbon nanorods and utilized them as MFC anode electrocatalysts. The high biocompatibility of MoO_2_ could enrich electroactive bacteria on the anode, and modification with Co augmented the electrocatalytic activity; simultaneously, N doping improved the electronic conductivity of the carbon nanorods. Their experimental results also indicate good electrocatalytic activity during charge transfer of the anode, and the maximum power density of the MFC is 2.06 ± 0.05 W m^−2^. A previous study discussed nitrogen-doped CC grafting with MoO_2_ microspheres (N@MoO_2_/CC) prepared via in situ polymerization and high-temperature carburization for the development of high-performance anodes of MFCs [41]. The N@MoO_2_/CC anode exhibited remarkable bioelectricity generation due to its dual function of promoting bacterial colonization and enriching electroactive bacteria, thereby improving the electrocatalytic activity of the anode. An MFC with the N@MoO_2_/CC anode obtained a maximum power density of 3.01 ± 0.23 W m^−2^. Furthermore, MWCNTs composites modified with MoO_2_ nanoparticles have been used for methanol oxidation [42]. MWCNTs@MoO_2_–C nanocable composites have outstanding electrochemical performance in lithium-ion battery anodes [43]. To the best of our knowledge, relevant systematic reports about MoO_2_/MWCNTs nanocomposites as anodes of *Escherichia coli* (*E. coli*)-inoculated MFCs are scarce.

According to the above studies, multi-walled CNTs (MWCNTs) have superior electronic conductivity and MoO_2_ nanoparticles have been confirmed to have excellent biocompatibility and electrocatalytic activity. We consider and propose the effective recombination of functionalized MWCNTs and MoO_2_ nanoparticles to construct anodic modification materials for MFCs. This can give full play to the advantages of functionalized MWCNTs and MoO_2_ nanoparticles. Accordingly, in this paper, MoO_2_-nanoparticle-decorated functionalized MWCNTs (MoO_2_/MWCNTs) nanocomposites are fabricated by mixing functionalized MWCNTs and phosphomolybdic acid in a tube furnace in a hydrogen–argon (10%) atmosphere. These nanocomposites are then utilized as anodic modification materials for *E. coli*-inoculated MFCs. Electrochemical cyclic voltammetry (CV) measurements show that the electroactive areas of the functionalized MWCNTs and MoO_2_/MWCNTs electrodes are notably enhanced compared with those of nonfunctionalized MWCNTs and bare CC electrodes. This is beneficial for the mass attachment of active bacteria and the rapid formation of stable biofilms. Moreover, the MoO_2_/MWCNTs anode has enhanced EET efficiency and nutrient transfer capability. Therefore, compared with the bare CC, MWCNT, and functionalized MWCNTs anodes, the MFC with the MoO_2_/MWCNTs nanocomposites bioanode has higher power density outputs and long-term voltage and current density stability. Herein, a carbon-based transition metal oxide nanocomposite (MoO_2_/MWCNTs) is successfully prepared and utilized as an MFC anode. This establishes a solid theoretical and experimental foundation for searching for anodic modification materials for high-performance MFCs.

## 2. Results and Discussion

### 2.1. Physical Characterization of Materials

**Figure 1:**
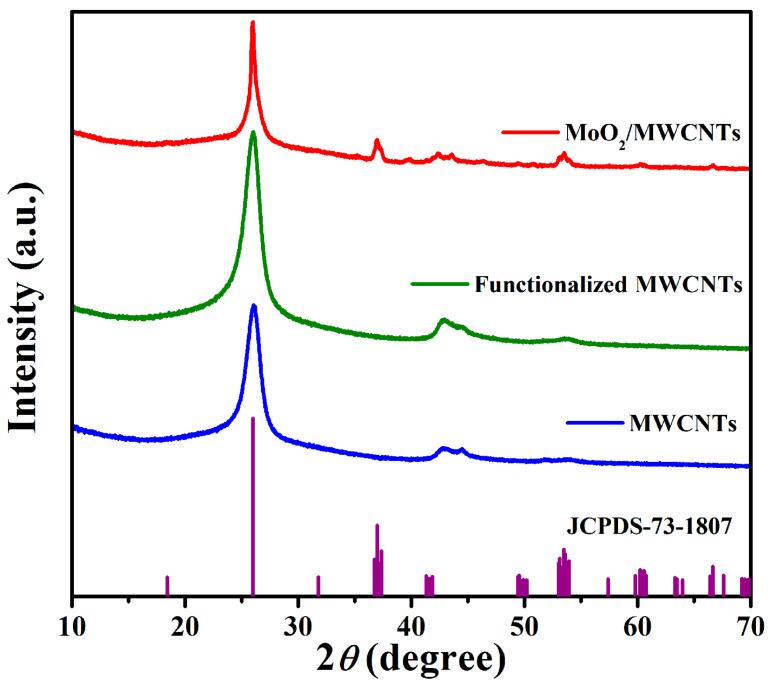
XRD patterns of MWCNTs, functionalized MWCNTs, and MoO_2_/MWCNTs nanocomposites.

X-ray diffraction (XRD) patterns can characterize the phase and crystallinity of materials. Figure 1 shows the XRD patterns of MWCNTs, functionalized MWCNTs, and the MoO_2_/MWCNTs nanocomposites. All three materials display strong diffraction peaks at approximately 2*θ* = 26°, which is a typical characteristic (002) diffraction peak of MWCNTs. Compared with the diffraction peak of the nonfunctionalized MWCNTs, that of the functionalized MWCNTs is slightly wider, and no other special diffraction peaks appear. Nevertheless, the MoO_2_/MWCNTs nanocomposites show MoO_2_ characteristic peaks at 2*θ* = 36.979°, 37.344°, 53.293°, 53.578°, 53.938°, 60.251°, and 66.653° (JCPDS-73-1807). This indicates the successful preparation of the MoO_2_/MWCNTs nanocomposite.

**Figure 2:**
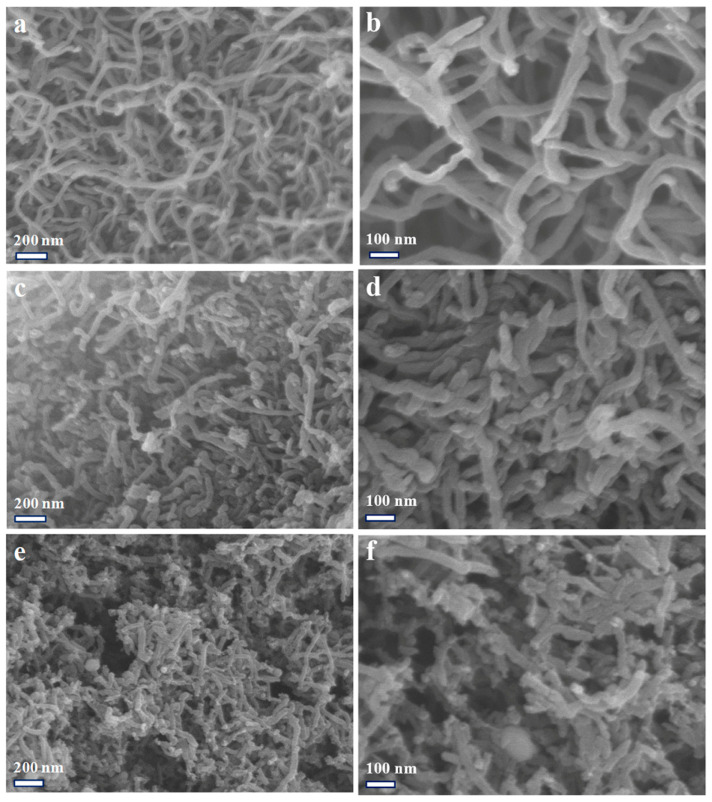
SEM images of (**a**,**b**) MWCNTs, (**c**,**d**) functionalized MWCNTs, and (**e**,**f**) MoO_2_/MWCNTs.

The surface morphologies of the MWCNTs, functionalized MWCNTs, and MoO_2_/MWCNTs are characterized using scanning electron microscopy (SEM). Figure 2a–f shows the SEM images of the MWCNTs, functionalized MWCNTs, and MoO_2_/MWCNTs magnified 50,000 and 100,000 times, respectively. As exhibited in Figure 2a, the MWCNTs have evident tubular structures. Figure 2b shows that the nanotubes form interlaced spatial network frameworks. The functionalized MWCNTs are short (Figure 2c) because ultrasound in mixed acids can separate nanotubes and generate oxygen-containing groups on MWCNTs surfaces [44]. As seen in Figure 2d, the wall surfaces of the functionalized MWCNTs are slightly rough, which may be due to the presence of surface oxygen-containing groups. Figure 2e,f depicts numerous small particles loaded on the MWCNTs. Combined with the XRD diagram of MoO_2_/MWCNTs, these figures indicate that these small particles are MoO_2_ nanoparticles. Figure 2f shows the formation of a spatial grid construction and large holes. These are subsequently verified using the SEM–EDS elemental analysis spectra of the MWCNTs, functionalized MWCNTs, and MoO_2_/MWCNTs in Figure S1. The pristine MWCNTs (Figure S1a) and functionalized MWCNTs (Figure S1b) have no Mo elements, whereas MoO_2_/MWCNTs have Mo elements (Figure S1c).

**Figure 3:**
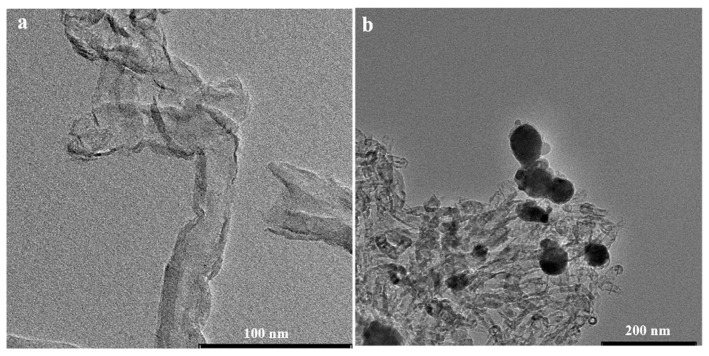
TEM images of (**a**) functionalized MWCNTs and (**b**) MoO_2_/MWCNTs.

The morphologies of the functionalized MWCNTs and the MoO_2_/MWCNTs nanocomposites are further characterized using transmission electron spectroscopy (TEM). As shown in Figure 3a,b, the functionalized MWCNTs (Figure 3a) have clear tubular configurations, and the nanotube surfaces have no granular materials. In contrast, numerous MoO_2_ nanoparticles are anchored on the functionalized MWCNTs in MoO_2_/MWCNTs nanocomposites (Figure 3b). The sizes of the MoO_2_ nanoparticles are not distributed homogeneously; they range from 20 to 60 nm, and a few nanoparticles are agglomerated.

**Figure 4:**
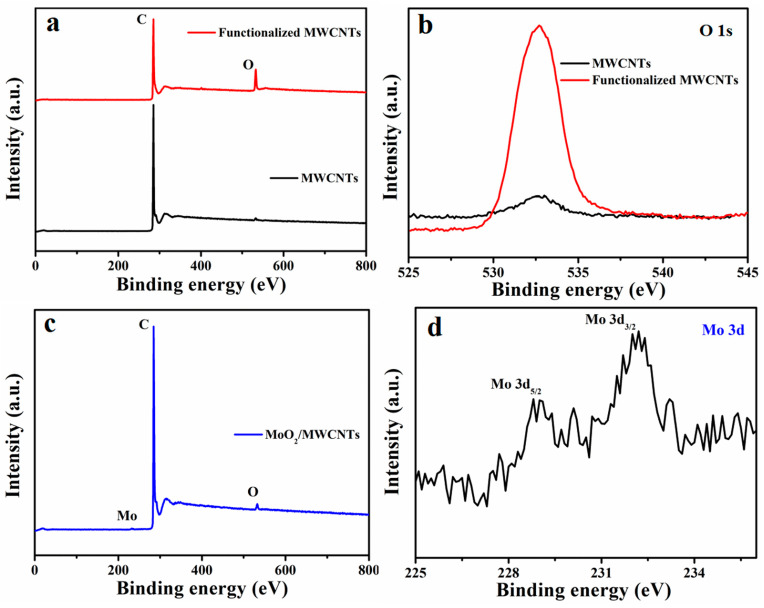
(**a**) Wide XPS spectra of MWCNTs and functionalized MWCNTs, (**b**) O 1s spectra of MWCNTs and functionalized MWCNTs, (**c**) wide XPS spectrum of MoO_2_/MWCNTs, and (**d**) Mo 3d spectrum of MoO_2_/MWCNTs.

The wide XPS spectra of the MWCNTs and functionalized MWCNTs are shown in Figure 4a. The nonfunctionalized MWCNTs have C and trace O, which may be caused by small amounts of oxygen-containing functional groups on the nanotube end caps of the MWCNTs or oxygen from the atmosphere. The functionalized MWCNTs also have C and O elements, but the O/C ratio is dramatically higher than that of the MWCNTs. In addition, the O 1s spectra of the MWCNTs and functionalized MWCNTs are shown in Figure 4b. The O area in the functionalized MWCNTs is pronouncedly larger than that of the pristine MWCNTs. These findings verify the successful functionalization of the MWCNTs via mixed-acid treatment. Figure 4c shows the wide XPS spectrum of MoO_2_/MWCNTs, which shows the existence of C, Mo, and O. Figure 4d presents the Mo 3d spectrum of MoO_2_/MWCNTs. Here, the electron binding energies at approximately 229.0 and 232.2 eV are assigned to the 3d_5/2_ and 3d_3/2_ components, respectively, of the Mo(IV) in MoO_2_, matching previous literature [40]. These again prove the successful fabrication of the MoO_2_/MWCNTs nanocomposites.

### 2.2. Electrochemical Behaviors of Electrodes

**Figure 5:**
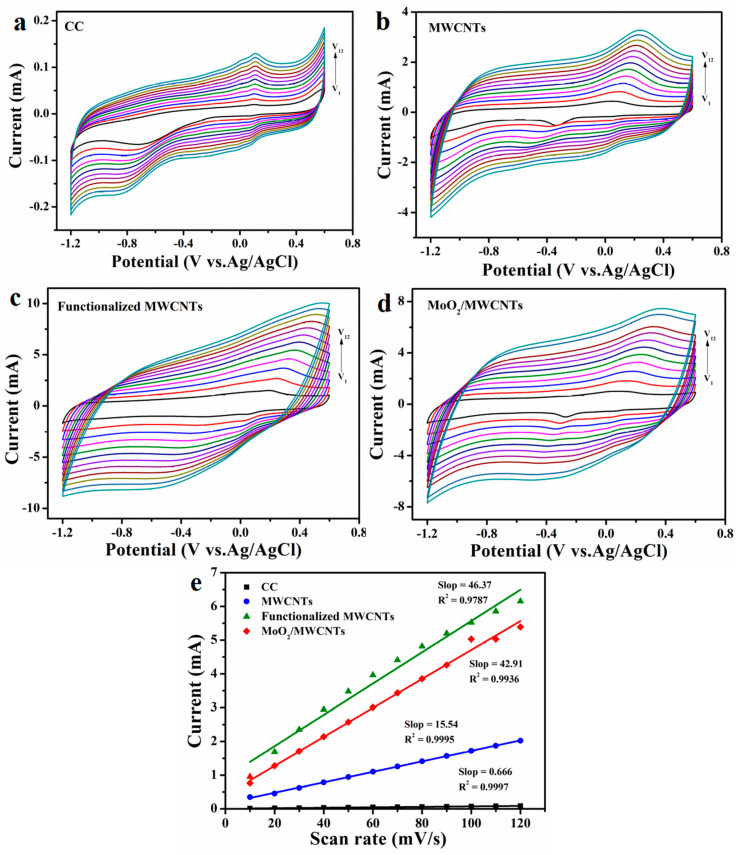
CV responses of electrodes in PBS solution at various scan rates: (**a**) bare CC, (**b**) MWCNTs, (**c**) functionalized MWCNTs, and (**d**) MoO_2_/MWCNTs. The curve numbers are the millivolts per second of (ν_1_–ν_12_) 10–120; (**e**) Evaluation of C_dl_ by plotting mean capacitance current values against corresponding scan rates.

Large electroactive areas in MFC anodes are favorable for the massive adherence of active bacteria; the electroactive areas of anodes are estimated via electrochemical double-layer capacitance [45,46]. The capacitance properties of MWCNTs, functionalized MWCNTs, and MoO_2_/MWCNTs electrodes with the same geometric areas are evaluated using their electrochemical CV behaviors. Figure 5a–d depicts the CV responses of bare CC, MWCNTs, functionalized MWCNTs, and MoO_2_/MWCNTs electrodes in a phosphate buffer saline (PBS) solution at scan rates of 10, 20, 30, 40, 50, 60, 70, 80, 90, 100, 110, and 120 mV s^−1^ (−1.2–0.6 V vs. Ag/AgCl). The double-layer capacitance (C_dl_) values (unit: mF) of the four electrodes are calculated using a previously reported formula [47,48]. The C_dl_ values of the bare CC, MWCNTs, functionalized MWCNTs, and MoO_2_/MWCNTs electrodes are 0.666, 15.54, 46.37, and 42.91 mF, respectively (Figure 5e). The C_dl_ of the bare CC electrode is extremely small. The C_dl_ values of the functionalized MWCNTs and MoO_2_/MWCNTs electrodes are 2.98 and 2.76 times, respectively, that of the pristine MWCNTs electrode. The larger C_dl_ of the functionalized MWCNTs and MoO_2_/MWCNTs electrodes implies that these electrodes have larger electrochemically active areas. Such electroactive areas help improve MFC performance [49]. However, anodes with large electroactive areas do not necessarily have high power densities. The power density of an MFC is also highly related to the electron conductivity of the anode, the EET rate between the anode and active microorganisms, the efficiency of nutrient transfer to the biofilm surface, and other factors [2,50,51]. The functionalized MWCNTs and MoO_2_/MWCNTs electrodes have large electroactive areas, indicating that the increase in the electroactive area of the MoO_2_/MWCNTs nanocomposite is mainly attributed to MWCNTs functionalization. Large numbers of components with oxygen-containing functional groups attach to the surfaces of functionalized MWCNTs; consequently, functionalized MWCNTs have little aggregation and easily disperse due to electrostatic interactions, and their active groups are easily exposed [52]. In addition, a large internal capacitance of an anode material can enhance its instantaneous charge storage, which is also important for improving MFC performance [46]. The CV curves of the MoO_2_/MWCNTs electrode form almost rectangular shapes in Figure 5d, suggesting that the MoO_2_/MWCNTs nanocomposite has high electronic conductivity [45]; this is due to the excellent electronic conductivity and charge transmission of the MoO_2_ nanoparticles in the MoO_2_/MWCNTs electrode [37,38].

### 2.3. Investigation of MFC Performance

**Figure 6:**
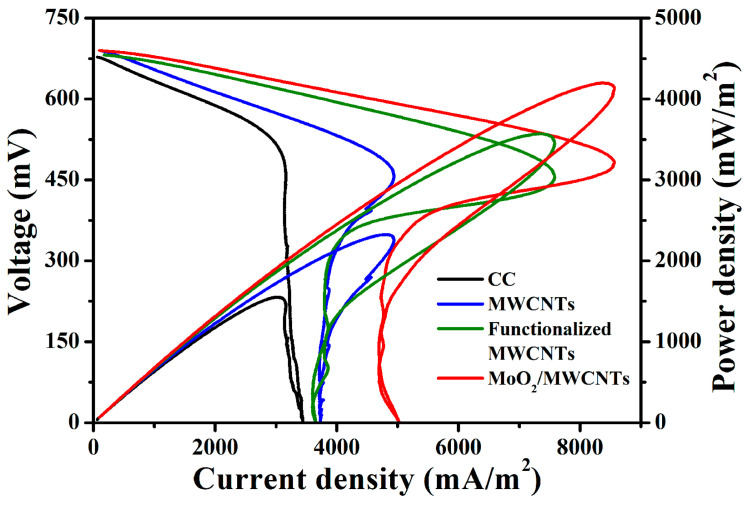
Power density and polarization curves of MFCs with bare CC, MWCNTs, functionalized MWCNTs, and MoO_2_/MWCNTs bioanodes at a scan rate of 1.0 mV s^−1^.

When the voltage outputs of the MFCs reach stable plateaus on a voltage test card, electrochemical linear sweep voltammetry (LSV) measurements are performed to collect the polarization curves of MFCs with bare CC, MWCNTs, functionalized MWCNTs, and MoO_2_/MWCNTs bioanodes. Their power outputs are recorded and evaluated using power (*P*) proportional to the geometrical area (*A*) of the anode (*P_A_* = *P*/*A*). The *p* values are calculated using voltages and homologous biocurrent values (*P* = *UI*) on the MFC polarization curves. The polarization and power density curves are used to assess the bioelectricity generation performance of *E. coli*-catalyzed MFCs. Figure 6 illustrates the power density and polarization curves of the MFCs with bare CC, MWCNT, functionalized MWCNTs, and MoO_2_/MWCNTs bioanodes at a scan rate of 1.0 mV s^−1^. The maximum power densities of the four MFCs are 1541.36, 2300.29, 3556.89, and 4185.20 mW m^−2^, respectively. The corresponding biocurrent densities are 3027.06, 4851.80, 7432.35, and 8466.50 mA m^−2^, respectively. Evidently, the maximum power and current density of the MFC with the MoO_2_/MWCNTs bioanode are superior to those of the MFCs with the bare CC, MWCNTs, and functionalized MWCNTs bioanodes. The maximum power density of the MFC with the MoO_2_/MWCNTs bioanode is 2.72-, 1.82-, and 1.18-fold higher than those of the MFCs with the bare CC, MWCNTs, and functionalized MWCNTs bioanodes, respectively. Hence, as an anodic modification material, the MoO_2_/MWCNTs nanocomposite can markedly enhance the power generation performance of MFCs. The *E. coli*-inoculated MFC with the functionalized MWCNTs anode in our study reached a maximum power density of 3556.89 mW m^−2^ at a scan rate of 1.0 mV s^−1^, which is slightly lower than that of an *E. coli*-inoculated MFC with a CNTs anodic electrocatalyst based on a PBE binder at a scan rate 1.0 mV s^−1^ (3800 mW m^−2^) [26]. Nevertheless, the MFC with the MoO_2_/MWCNTs anode yields a higher power density of 4185.20 mW m^−2^. Thus, the introduction of MoO_2_ nanoparticles into the MoO_2_/MWCNTs anode can enhance the power generation of MFCs. This may be attributed to the large electroactive area and high electronic conductivity of the MoO_2_/MWCNTs anode.

**Figure 7:**
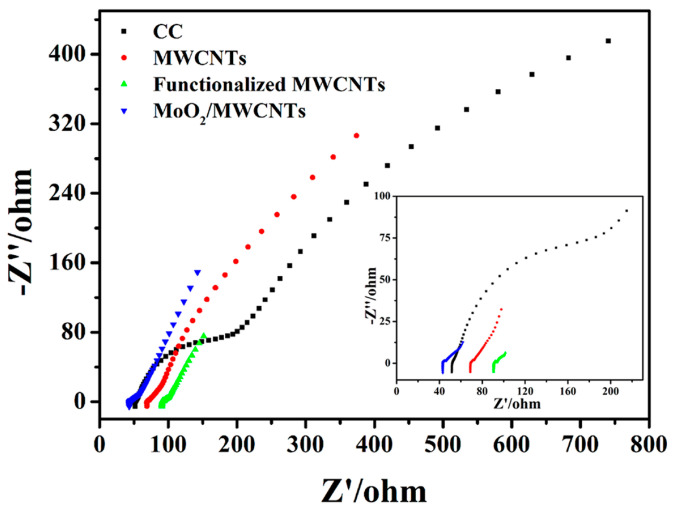
EIS spectra of bare CC, MWCNTs, functionalized MWCNTs, and MoO_2_/MWCNTs bioanodes at frequencies of 100 kHz–10 mHz and an amplitude of 5 mV.

The electrochemical impedance spectroscopy (EIS) spectra of the bare CC, MWCNTs, functionalized MWCNTs, and MoO_2_/MWCNTs bioanodes are shown in Figure 7. The semicircle in the high-frequency region of the EIS spectra shows the charge transfer impedance (R_ct_) from the electroactive exoelectrogens to the anodic electrocatalyst [45]. The bare CC bioanode has the largest R_ct_, confirming the poor EET rate and high cell internal resistance of the MFC with the bare CC bioanode. The MWCNTs, functionalized MWCNTs, and MoO_2_/MWCNTs bioanodes have relatively smaller R_ct_ values, reflecting the accelerated EET from the electroactive microbes to anodic modification and the smaller cell internal resistance of the MFCs [51,53]. In Figure 7, the first intersection point of the EIS curves and the *x* axis is the impedance of the anodic solution (R_s_). A smaller R_s_ value indicates faster interfacial mass transfer between the anodic solution and the bioanode, indicating that more nutrients in the anodic solution are used for active *E. coli* growth. Nutrient transfer to the biofilm surface is the primary driver of MFC energy production [51]. The MoO_2_/MWCNTs bioanode has the lowest R_s_ value, suggesting better interface mass transfer efficiency between the MoO_2_/MWCNTs bioanode and the anodic solution, which is essential for improving MFC performance. The MoO_2_/MWCNTs nanocomposite integrates the synergistic advantages of the functionalized MWCNTs and MoO_2_ nanoparticles, facilitating nutrient transfer to the biofilm surface on the MoO_2_/MWCNTs anode and achieving superior power density.

### 2.4. Discharge Repeatability

**Figure 8:**
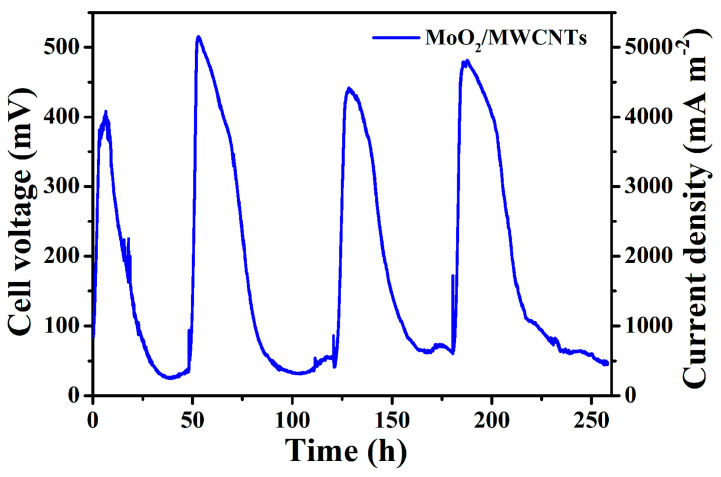
Cell voltage and current density profiles over four consecutive discharge cycles for MFC with the MoO_2_/MWCNTs bioanode.

Figure 8 shows the cell voltage and current density profiles over four continuous discharge cycles of the MFC with the MoO_2_/MWCNTs bioanode. The voltage peak of the MFC in the first discharge cycle emerges at approximately 400 mV, and the corresponding current density peak is approximately 4000 mA m^−2^. The voltage plateaus reach approximately 510, 450, and 480 mV from the second discharge cycle to the fourth, whereas the corresponding current densities reach approximately 5100, 4500, and 4800 mA m^−2^, respectively. This may be attributed to the stimulated electrochemical activation of the electroactive bacteria (*E. coli*) on the anode surface during long-term MFC operation [54]. Consequently, enhanced voltage and current density plateaus appear in the second to the fourth discharge cycles of the MFC. These findings indicate the satisfactory electricity generation capacity and long-term voltage-output repeatability of the MFC with the MoO_2_/MWCNTs bioanode.

## 3. Experimental Section

### 3.1. Fabrication and Characterization of Materials

The fabrication procedures of the MoO_2_/MWCNTs nanocomposites are as follows: first, MWCNTs are functionalized through mixed-acid treatment. Then, 1.0 g of MWCNTs powder is placed in a 200 mL beaker and a 40 mL mixture of concentrated nitric acid and sulfuric acid (volume ratio: 1:3) is slowly poured into the beaker. The beaker is sealed using a sealing film, placed under ultrasound for 1 h, and left to sit at room temperature for 24 h. The mixture is pumped and filtered, washed with ultrapure water (ρ = 18.25 MΩ cm) several times until the pH value is close to neutral, and vacuum-dried at 60 °C for later use. Second, 100 mg of functionalized MWCNTs are added to a 50 mL phosphomolybdic acid aqueous solution with a concentration of 20 mg mL^−1^. After 12 h of magnetic stirring, the suspension is centrifuged at 10,000 rpm min^−1^ and then washed with ultrapure water three times following cryogenic desiccation. The aforementioned samples are placed in a tube furnace, where a hydrogen–argon (10%) mixture is exhausted for 30 min in advance. The samples are then subjected to reduction at 900 °C for 3 h. MoO_2_/MWCNTs nanocomposite powder is acquired after the mixture is allowed to naturally cool to normal temperature.

The characterization details of the materials are in the Supplementary Materials.

### 3.2. Electrode Construction

CCs (1.0 cm × 1.0 cm) (331P, Hesen, Shanghai, China) are pretreated by sequentially dipping them in KOH (1.0 M), ultrapure water, HCl (1.0 M), and ultrapure water for 1 h and then vacuum-dried for 8 h at 60 °C. The pretreated CCs are connected using fine copper wires, and parts with copper leakage are wrapped using AB glue (Deli, Ningbo, China). Carbon papers (CPs, 2.0 cm × 2.0 cm) (030P, Hesen, Shanghai, China) are pretreated following the above procedure. The prepared CC electrode (1.0 cm × 1.0 cm) is utilized as an anode substrate electrode (Figure S2).

Next, 2.0 mg of the MoO_2_/MWCNTs powder is mixed with 400 μL of Nafion–alcohol solution (0.1 wt%), and a uniform suspension forms after ultrasound exposure for 1 h. The suspension is added drop by drop to the prepared CC surface (1.0 cm × 1.0 cm), dried under infrared light, and used as an MFC anode. The MWCNTs, functionalized MWCNTs, and modified CC electrodes are prepared following the above process. MWCNTs, functionalized MWCNTs, and bare CC electrodes (1.0 cm × 1.0 cm) are the contrast anodes.

Bare pretreated CP electrodes (2.0 cm × 2.0 cm) are the cathodes of all MFCs.

### 3.3. Establishment, Operation, and Evaluation of E. coli-Inoculated MFCs

Cubic dual-chamber MFCs (volume of single chamber: 100 mL) with proton exchange membranes (Nafion 212, Dupont, Wilmington, DE, USA) are used to assess the performance of the MFCs with the bare CC, MWCNTs, functionalized MWCNTs, and MoO_2_/MWCNTs bioanodes. The microorganism *E. coli* (BNCC133264, Beijing, China) in a fluid nutrient medium (nutrient broth, Aobox, Beijing, China) is placed in a thermostatic incubator for 20 h at 37 °C. PBS solutions (pH = 7.0) are used as the base electrolyte of the anodes and cathodes; it comprises NaH_2_PO_4_⋅2H_2_O (11.05 g L^−1^) and NaHCO_3_ (10.0 g L^−1^). A 50 mL PBS solution containing 5.0 g L^−1^ yeast extract, 10.0 g L^−1^ glucose, and 5 mM 2-hydroxy-1, 4-naphthoquinone (HNQ, Sigma–Aldrich, St. Louis, MO, USA) is utilized as the MFC anolyte. Next, 20 mL of fluid nutrient media containing *E. coli* is inoculated into the anode chambers and utilized as active microflora biocatalysts of the MFCs. The catholyte is a 70 mL PBS solution including 0.1 M KCl and 50 mM K_3_[Fe(CN)_6_]. All items are sterilized at 121 °C in an autoclave for 15–20 min in advance.

High-purity nitrogen is pumped into the anode chambers of the MFCs for 50 min to remove the dissolved oxygen from the anolyte and domesticate *E. coli* in the anaerobic atmospheres. Resistors (1000 Ω) are connected to external circuits. Next, the MFCs are placed in a thermostatic water bath at 37 °C. The voltages across the resistor are collected using a NI6009 voltage test card (NI, USA). Electrochemical measurements are obtained using a CHI760e workstation (Chenhua, Shanghai, China). The polarization curves of the MFCs with the bare CC, MWCNTs, functionalized MWCNTs, and MoO_2_/MWCNTs bioanodes are acquired via electrochemical LSV (scanning interval: open-circuit potential to 0 V). EIS is performed at the open-circuit potential (amplitude: 5 mV; frequency domain: 10^5^–10^−2^ Hz). The MFC bioanodes are the working electrodes and the MFC cathodes are the reference and counter electrodes.

## 4. Conclusions

Herein, we successfully construct a MoO_2_/MWCNTs nanocomposite by mixing functionalized MWCNTs and phosphomolybdic acid in a tube furnace in a hydrogen–argon (10%) atmosphere. Its composition and morphology are studied using XRD, SEM, TEM, and XPS. MWCNTs, functionalized MWCNTs, and MoO_2_/MWCNTs materials are utilized as anodic modification materials for *E. coli*-inoculated MFCs. Abundant oxygen-containing groups attach to the surfaces of the functionalized MWCNTs, which can result in large electroactive areas and is conducive to the attachment of active bacteria. A larger internal capacitance of anode materials can enhance instantaneous charge storage, which is also important for improving MFC performance. Combining the large electroactive areas of the functionalized MWCNTs and the outstanding charge transmission of the MoO_2_ nanoparticles, the MoO_2_/MWCNTs anode has a large electroactive area and excellent electronic conductivity. Additionally, the MFC with the MoO_2_/MWCNTs bioanode exhibits a small charge transfer impedance and anodic solution impedance, demonstrating the efficient EET and good nutrient transfer capability of the bioanode. Finally, the MFC with the MoO_2_/MWCNTs bioanode generates high power density (2.72-, 1.82-, and 1.18-fold higher than those of the MFCs with the bare CC, nonfunctionalized MWCNTs, and functionalized MWCNTs bioanodes, respectively). This paper can be a reference for further applications of carbon-based transition metal oxide nanocomposites in the field of high-performance MFCs.

## Data Availability

Data are contained within the article and Supplementary Materials.

## Supplementary Materials

The following supporting information can be downloaded at: https://www.mdpi.com/article/10.3390/molecules29112541/s1, Figure S1: EDS elemental analysis spectrums of (a) MWCNTs; (b) Functionalized MWCNTs and (c) MoO_2_/MWCNTs materials. Figure S2: Digital photo of fabricated CC substrate electrode.

## References

[1] Feng M., Meng L., Zhang Z., Zheng Q., Wang R., Yang C., Guo W. (2024). Hierarchical modulation of extracellular electron transfer processes in microbial fuel cell anodes for enhanced power output through improved Geobacter adhesion. Electrochim. Acta.

[2] Zou L., Huang Y.H., Wu X., Long Z.E. (2019). Synergistically promoting microbial biofilm growth and interfacial bioelectrocatalysis by molybdenum carbide nanoparticles functionalized graphene anode for bioelectricity production. J. Power Sources.

[3] Slate A.J., Whitehead K.A., Brownson D.A., Banks C.E. (2019). Microbial fuel cells: An overview of current technology. Renew. Sustain. Energy Rev..

[4] Kumar G.G., Awan Z., Nahm K.S., Xavier J.S. (2014). Nanotubular MnO_2_/graphene oxide composites for the application of open air-breathing cathode microbial fuel cells. Biosens. Bioelectron..

[5] Chouler J., Padgett G.A., Cameron P.J., Preuss K., Titirici M.-M., Ieropoulos I., Di Lorenzo M. (2016). Towards effective small scale microbial fuel cells for energy generation from urine. Electrochim. Acta.

[6] Wang Y., Zhong K., Li H., Dai Y., Zhang H., Zuo J., Yan J., Xiao T., Liu X., Lu Y. (2021). Bimetallic hybrids modified with carbon nanotubes as cathode catalysts for microbial fuel cell: Effective oxygen reduction catalysis and inhibition of biofilm formation. J. Power Sources.

[7] Ma J.C., Zhang J., Zhang Y.Z., Guo Q.L., Hu T.J., Xiao H., Jia J.F. (2023). Progress on anodic modification materials and future development directions in microbial fuel cells. J. Power Sources.

[8] Rossi R., Cario B.P., Santoro C., Yang W., Saikaly P.E., Logan B.E. (2019). Evaluation of electrode and solution area-based resistances enables quantitative comparisons of factors impacting microbial fuel cell performance. Environ. Sci. Technol..

[9] Senthilkumar N., Pannipara M., Al-Sehemi A.G., Kumar G.G. (2019). PEDOT/NiFe_2_O_4_ nanocomposites on biochar as a free-standing anode for high-performance and durable microbial fuel cells. New J. Chem..

[10] Marks S., Makinia J., Fernandez-Morales F.J. (2019). Performance of microbial fuel cells operated under anoxic conditions. Appl. Energy.

[11] Li B., Zhou J., Zhou X., Wang X., Li B., Santoro C., Grattieri M., Babanova S., Artyushkova K., Atanassov P. (2014). Surface modification of microbial fuel cells anodes: Approaches to practical design. Electrochim. Acta.

[12] Gnana Kumar G., Kirubaharan C.J., Udhayakumar S., Ramachandran K., Karthikeyan C., Renganathan R., Nahm K.S. (2014). Synthesis, Structural, and Morphological Characterizations of Reduced Graphene Oxide-Supported Polypyrrole Anode Catalysts for Improved Microbial Fuel Cell Performances. ACS Sustain. Chem. Eng..

[13] Babanova S., Jones J., Phadke S., Lu M., Angulo C., Garcia J., Carpenter K., Cortese R., Chen S., Phan T.T. (2020). Continuous flow, large-scale, microbial fuel cell system for the sustained treatment of swine waste. Water Environ. Res..

[14] Li F., Wang D., Liu Q., Wang B., Zhong W., Li M., Liu K., Lu Z., Jiang H., Zhao Q. (2019). The construction of rod-like polypyrrole network on hard magnetic porous textile anodes for microbial fuel cells with ultra-high output power density. J. Power Sources.

[15] Yu B., Li Y., Feng L. (2019). Enhancing the performance of soil microbial fuel cells by using a bentonite-Fe and Fe_3_O_4_ modified anode. J. Hazard. Mater..

[16] Wang R., Liu D., Yan M., Zhang L., Chang W., Sun Z., Liu S., Guo C. (2019). Three-dimensional high performance free-standing anode by one-step carbonization of pinecone in microbial fuel cells. Bioresour. Technol..

[17] Gnana Kumar G., Kirubaharan C.J., Yoo D.J., Kim A.R. (2016). Graphene/poly(3,4-ethylenedioxythiophene)/Fe_3_O_4_ nanocomposite—An efficient oxygen reduction catalyst for the continuous electricity production from wastewater treatment microbial fuel cells. Int. J. Hydrogen Energy.

[18] Catal T., Kul A., Atalay V.E., Bermek H., Ozilhan S., Tarhan N. (2019). Efficacy of microbial fuel cells for sensing of cocaine metabolites in urine-based wastewater. J. Power Sources.

[19] Liu S.-H., Lai Y.-C., Lin C.-W. (2019). Enhancement of power generation by microbial fuel cells in treating toluene-contaminated groundwater: Developments of composite anodes with various compositions. Appl. Energy.

[20] Ren P., Ci S., Ding Y., Wen Z. (2019). Molten-salt-mediated synthesis of porous Fe-containing N-doped carbon as efficient cathode catalysts for microbial fuel cells. Appl. Surf. Sci..

[21] Kirubaharan C.J., Kumar G.G., Sha C., Zhou D., Yang H., Nahm K.S., Raj B.S., Zhang Y., Yong Y.-C. (2019). Facile fabrication of Au@polyaniline core-shell nanocomposite as efficient anodic catalyst for microbial fuel cells. Electrochim. Acta.

[22] Zhang K., Ma Z., Li X., Zhang M., Song H. (2021). Good microbial affinity of phenolic carbon felt as an efficient anode for microbial fuel cells. Bioelectrochemistry.

[23] Muthukumar H., Mohammed S.N., Chandrasekaran N., Sekar A.D., Pugazhendhi A., Matheswaran M. (2019). Effect of iron doped zinc oxide nanoparticles coating in the anode on current generation in microbial electrochemical cells. Int. J. Hydrogen Energy.

[24] Mehdinia A., Ziaei E., Jabbari A. (2014). Multi-walled carbon nanotube/SnO_2_ nanocomposite: A novel anode material for microbial fuel cells. Electrochim. Acta.

[25] Mohan Y., Das D. (2009). Effect of ionic strength, cation exchanger and inoculum age on the performance of microbial fuel cells. Int. J. Hydrogen Energy.

[26] Li H., Liao B., Xiong J., Zhou X., Zhi H., Liu X., Li X., Li W. (2018). Power output of microbial fuel cell emphasizing interaction of anodic binder with bacteria. J. Power Sources.

[27] Tsai H.-Y., Wu C.-C., Lee C.-Y., Shih E.P. (2009). Microbial fuel cell performance of multiwall carbon nanotubes on carbon cloth as electrodes. J. Power Sources.

[28] Sun J.-J., Zhao H.-Z., Yang Q.-Z., Song J., Xue A. (2010). A novel layer-by-layer self-assembled carbon nanotube-based anode: Preparation, characterization, and application in microbial fuel cell. Electrochim. Acta.

[29] Peng L., You S.-J., Wang J.-Y. (2010). Carbon nanotubes as electrode modifier promoting direct electron transfer from shewanella oneidensis. Biosens. Bioelectron..

[30] Liang P., Wang H., Xia X., Huang X., Mo Y., Cao X., Fan M. (2011). Carbon nanotube powders as electrode modifier to enhance the activity of anodic biofilm in microbial fuel cells. Biosens. Bioelectron..

[31] Liu X.-W., Huang Y.-X., Sun X.-F., Sheng G.-P., Zhao F., Wang S.-G., Yu H.-Q. (2014). Conductive carbon nanotube hydrogel as a bioanode for enhanced microbial electrocatalysis. ACS Appl. Mater. Interfaces.

[32] Hirsch A., Vostrowsky O. (2005). Functionalization of carbon nanotubes. ChemInform.

[33] Tasis D., Tagmatarchis N., Georgakilas V., Pantarotto D., Vaccari L., Bianco A., Guldi D.M., Prato M. (2003). Organic functionalization of carbon nanotubes. ChemInform.

[34] Liu Y., Yu G., Li G.D., Sun Y., Asefa T., Chen W., Zou X. (2015). Coupling Mo_2_C with nitrogen-rich nanocarbon leads to efficient hydrogen-evolution electrocatalytic sites. Angew. Chem..

[35] Cui Z., Gong C., Guo C.X., Li C.M. (2013). Mo_2_C/CNTs supported Pd nanoparticles for highly efficient catalyst towards formic acid electrooxidation. J. Mater. Chem. A.

[36] Mai E.F., Machado M.A., Davies T.E., Lopez-Sanchez J.A., da Silva V.T. (2014). Molybdenum carbide nanoparticles within carbon nanotubes as superior catalysts for γ-valerolactone production via levulinic acid hydrogenation. Green Chem..

[37] Scanlon D.O., Watson G.W., Payne D.J., Atkinson G.R., Law D.S.L. (2010). Theoretical and experimental study of the electronic structures of MoO_3_ and MoO_2_. J. Phys. Chem. C.

[38] Shi Y., Guo B., Corr S.A., Shi Q., Hu Y.-S., Heier K.R., Chen L., Seshadri R., Stucky G.D. (2009). Ordered mesoporous metallic MoO_2_ materials with highly reversible lithium storage capacity. Nano Lett..

[39] Zeng L., Chen X., Li H., Xiong J., Hu M., Li X., Li W. (2018). Highly dispersed polydopamine-modified Mo_2_C/MoO_2_ nanoparticles as anode electrocatalyst for microbial fuel cells. Electrochim. Acta.

[40] Li L.X., Hu M., Zeng L., Xiong J., Tang B., Hu Z., Xing L., Huang Q., Li W. (2019). Co-modified MoO_2_ nanoparticles highly dispersed on N-doped carbon nanorods as anode electrocatalyst of microbial fuel cells. Biosens. Bioelectron..

[41] Hu F.M., Qiu Z.H., Zhang Z.Q., Zheng J.Y., He L.J., Gao H.P., Lin C.G. (2022). In-situ growth of N@MoO_2_ microflowers on carbon cloth for high-performance anodes in microbial fuel cells. J. Environ. Chem. Eng..

[42] Gao X.T., Wang Y.F., Fu L., Zhang R.X., Li R.M., Gao Z.H., Yan Z.F., Liu Y.M., Huang W., Liu L. (2023). High-performance Mo_2_C/MWCNT electrocatalyst for MOR: Comparison with MoO_2_/MWCNT and MoO_3_/MWCNT. Int. J. Hydrogen Energy.

[43] Guan J.P., Zhao L.P., Xing C.Y., Li Y.J. (2015). Novel flexible MWCNTs@MoO_2_-C nanocable composites with excellent electrochemical performance for lithium ion battery anodes. Mater. Res. Express.

[44] Liu Y., Gao L. (2005). A study of the electrical properties of carbon nanotube-NiFe_2_O_4_ composites: Effect of the surface treatment of the carbon nanotubes. Carbon.

[45] Xiong J., Hu M., Li X., Li H., Li X., Liu X., Cao G., Li W. (2018). Porous graphite: A facile synthesis from ferrous gluconate and excellent performance as anode electrocatalyst of microbial fuel cell. Biosens. Bioelectron..

[46] Tang J., Chen S., Yuan Y., Cai X., Zhou S. (2015). In situ formation of graphene layers on graphite surfaces for efficient anodes of microbial fuel cells. Biosens. Bioelectron..

[47] Mo C., Jian J., Li J., Fang Z., Zhao Z., Yuan Z., Yang M., Zhang Y., Dai L., Yu D. (2018). Boosting water oxidation on metal-free carbon nanotubes via directional interfacial charge-transfer induced by adsorbed polyelectrolyte. Energy Environ. Sci..

[48] Ma J., Shi N., Zhang Y., Zhang J., Hu T., Xiao H., Tang T., Jia J. (2020). Facile preparation of polyelectrolyte-functionalized reduced graphene oxide for significantly improving the performance of microbial fuel cells. J. Power Sources.

[49] Sevilla M., Mokaya R. (2014). Energy storage applications of activated carbons: Supercapacitors and hydrogen storage. Energy Environ. Sci..

[50] Wang R., Yan M., Li H., Zhang L., Peng B., Sun J., Liu D., Liu S. (2018). FeS_2_ nanoparticles decorated graphene as microbial-fuel-cell anode achieving high power density. Adv. Mater..

[51] Bian B., Shi D., Cai X., Hu M., Guo Q., Zhang C., Wang Q., Sun A.X., Yang J. (2018). 3D printed porous carbon anode for enhanced power generation in microbial fuel cell. Nano Energy.

[52] Liu J., Rinzler A.G., Dai H.J., Hafner J.H., Bradley R.K., Boul P.J., Lu A., Iverson T., Shelimov K., Huffman C.B. (1998). Fullerene pipes. Science.

[53] Ma J., Shi N., Jia J. (2020). Fe_3_O_4_ nanospheres decorated reduced graphene oxide as anode to promote extracellular electron transfer efficiency and power density in microbial fuel cells. Electrochim. Acta.

[54] Zhang T., Cui C., Chen S., Ai X., Yang H., Ping S., Peng Z. (2006). A novel mediatorless microbial fuel cell based on direct biocatalysis of *Escherichia coli*. Chem. Commun..

