# Supplementary material for: Fabrication of a Molybdenum Dioxide/Multi-Walled Carbon Nanotubes Nanocomposite as an Anodic Modification Material for High-Performance Microbial Fuel Cells
Source: Molecules. 2024 May 28;29(11):2541. doi: 10.3390/molecules29112541 (PMC11173943; doi:10.3390/molecules29112541)
Supplement: Supplementary file 1 [file molecules-29-02541-s001.zip › molecules-2990988-supplementary.pdf]

Supplementary Materials

Fabrication of a Molybdenum Dioxide/Multi-Walled Carbon Nanotubes Nanocomposite as an Anodic Modification Material for High-Performance Microbial Fuel Cells

Jianchun Ma [1,2,*], Lifang Wang [1,2], Yezhen Zhang [3] and Jianfeng Jia [4,*]

[1] Department of Chemical and Material Engineering, Lyuliang University, Lishi 033001, China; lllswlf@163.com
[2] Institute of New Carbon-Based Materials and Zero-Carbon and Negative-Carbon Technology, Lyuliang University, Lishi 033001, China
[3] College of Chemistry and Pharmacy Engineering, Nanyang Normal University, Nanyang 473061, China; zhangyezhenfang@sina.com
[4] Key Laboratory of Magnetic Molecules and Magnetic Information Materials of Ministry of Education, School of Chemistry and Materials Science, Shanxi Normal University, Taiyuan 030031, China
* Correspondence: singermajianchun@126.com (J.M.); jiajf@dns.sxnu.edu.cn (J.J.)

3.1. Synthesis and characterization of materials

X-ray diffraction (XRD) patterns of MWCNTs, functionalized MWCNTs and MoO_2/MWCNTs powders are acquired on the X-ray powder diffractometer (Ultima IV-185) with Cu Kα radiation. Morphologies of MWCNTs, functionalized MWCNTs and MoO_2/MWCNTs materials are characterized by scanning electron microscopy (SEM, JSM-7500F) and transmission electron microscopy (TEM, JEM-2100). X-ray photoelectron spectroscopy (XPS) is obtained by X-ray photoelectron spectroscope (Thermo Fisher K-AlPHA$^+$) with Al Kα radiation.

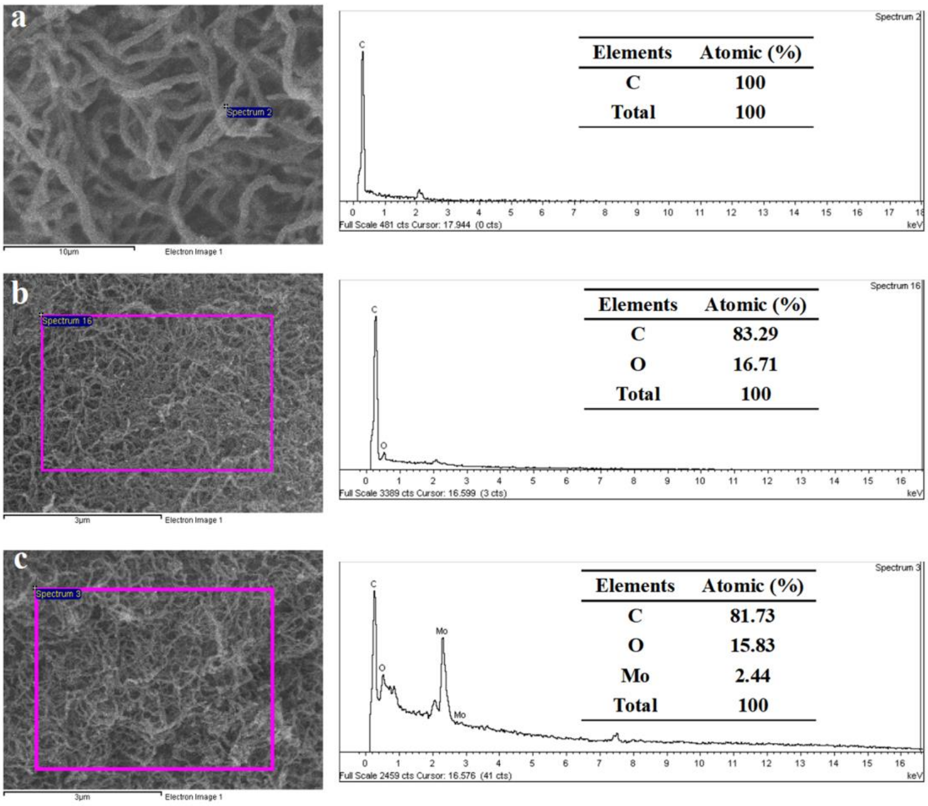

Figure S1. EDS elemental analysis spectrums of (a) MWCNTs; (b) Functionalized MWCNTs and (c) MoO_2/MWCNTs materials.

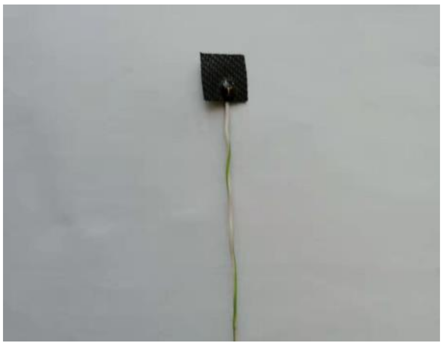

Figure S2. Digital photo of fabricated CC substrate electrode.
